# Preliminary study on carprofen concentration measurements after transcutaneous treatment with Vetdrop® in a microfracture joint defect model in sheep

**DOI:** 10.1186/s12917-014-0268-6

**Published:** 2014-12-09

**Authors:** Michèle Sidler, Nathalie Fouché, Ingmar Meth, Friedrich von Hahn, Brigitte von Rechenberg, Peter W Kronen

**Affiliations:** Musculoskeletal Research Unit (MSRU), Equine Hospital, Vetsuisse Faculty, University of Zurich, Zurich, Switzerland; Veterinary Anaesthesia Services International, Winterthur, Switzerland; Meddrop Technology AG, Thundorf, Switzerland; Competence Center for Applied Biotechnology and Molecular Medicine (CABMM), University of Zurich, Zurich, Switzerland

**Keywords:** Carprofen concentration measurements, Transcutaneous treatment, Sheep

## Abstract

**Background:**

The present preliminary study describes concentration time courses of the NSAID carprofen in the plasma and synovial fluid in a microfrature sheep model after transcutaneous treatments with a novel application device (Vetdrop®). To treat circumscribed inflammatory processes a transcutaneous application device could potentially be beneficial. After transcutaneous application normally lower systemic concentrations are measured which may reduce the incidence of side effects, whereas efficacy is still maintained.

In this study carprofen was used based on its capacity to provide analgesia after orthopaedic procedures in sheep and it is considered that it may have a positive influence on the healing of cartilage in low concentrations.

**Results:**

In all transcutaneously treated animals, carprofen plasma concentrations exceeded those of synovial fluid, although plasma levels remained significantly reduced (300-fold) as compared to carprofen administered intravenously. Furthermore, in contrast to the intravenously treated animals, a modest accumulation of carprofen in plasma and synovial fluid was observed in the transcutaneously treated animals over the 6-week treatment period.

**Conclusions:**

The transcutaneously administered carprofen using the Vetdrop® device penetrated the skin and both, plasma- and synovial concentrations could be measured repeatedly over time. This novel device may be considered a valuable transcutaneous drug delivery system.

## Background

Nonsteroidal anti-inflammatory drugs (NSAID’s) are commonly used in human and veterinary medicine to treat inflammatory processes and to relieve mild to moderate pain. Most NSAIDs inhibit the activity of the cyclooxygenase enzymes (COX1 and COX2), which produce prostaglandins. Prostaglandins are important factors for the pathogenesis of inflammation, swelling, pain and fever [1–3]. These prostaglandins also fulfil various physiological functions, such as mucosal defence through prevention and promotion of healing of mucosal erosions. The administration of NSAID’s can be accompanied by miscellaneous side effects, ranging from gastrointestinal mucosal damage, aplastic anaemia, and inhibition of thrombocyte aggregation to increased cardiovascular risks and renal failure [4–7]. Transcutaneous application of NSAID’s could potentially be beneficial when treating circumscribed inflammatory processes, due to the fact that systemic concentrations of the drugs are lower, thereby reducing the incidence of side effects. This concept is supported by findings from previous studies where the topical application of NSAID’s led to lower plasma levels as compared to systemic administration, whilst still maintaining efficacy [8–10].

The skin constitutes a barrier for nocive influences and therapeutic agents. Therefore, much effort has been invested into overcoming this barrier for therapeutic purposes. As such, the development of carriers to aid in the transport of therapeutic compounds across such barriers represents a possible way of skin penetration [11].

MedDrop Technology AG (Thundorf, Switzerland) has developed a treatment system (Vetdrop®) employing carriers for transcutaneous application of pharmaceutical agents, including the NSAID carprofen.

Carprofen was used in this study based on its capacity to provide analgesia after orthopaedic procedures [12,13] in sheep. In addition, carprofen may have a positive influence on the healing of cartilage in low concentrations [14]. Benton et al. [15] showed in an in vitro study that the administration of 1 and 10 μg/ml carprofen resulted in significantly higher glycosaminoglycan synthesis and cartilage matrix production, whereas a concentration of 20 μg/ml carprofen blocked this effect.

The current study was based on the hypothesis that the Vetdrop® Technology would allow the transcutaneous transport of carprofen into the stifle joint, which should be measureable in synovial fluid samples after application.

## Methods

The study was conducted according to the Swiss legal requirements for animal protection and welfare (TschG 455) and received ethical approval by the federal veterinary authorities ‘Kantonale Tierversuchskommission Zürich’ (permission No 193/2009). It was part of a larger-scale project published and described elsewhere [16]. In brief, 28 sheep with a mean age of 2.5 ± 0.5 years and a body weight average of 59.4 ± 10.2 kg underwent microfracture surgery on the medial condyle either on the left or right stifle joint.

The animals were allocated to 6 different treatment groups. Groups 1 – 5 were treated using the Vetdrop® system and group 6 received carprofen intravenously. The transcutaneous treatment system (Vetdrop®) was developed for the transcutaneous application of pharmaceutical substances. The system consists of an oxygen generator and an application system, which is used in conjunction with specially developed vehicle solutions. The oxygen generator extracts oxygen from the atmosphere and this highly concentrated oxygen serves as a propellant. The oxygen is first stored in a pressure container and during treatment, the oxygen flows through a pressure reducing valve and a treatment tube to the application device.

The applicator serves as a nano-dispersion-device consisting of a drug reservoir, which lies within a gas tank. The pharmaceutical ingredients are filled into the drug reservoir through a port, surrounded by the oxygen gas tank. The oxygen combines with carrier substance under pressure and propels it through the diffuser. The diffuser assembly utilizes the Venturi-Effect (fluid pressure decreases in response to a constricted area of flow), in order to disperse the carrier. The size of the droplets lies in the range of nanometers. The size is regulated via a needle lace, which alters the width of the port.

The employed carriers are a proprietary product of Arvine Pharma AG (MedVital Serum, Arvine Pharma AG, Thundorf Switzerland). The vehicles are based on oil-in-water or water-in-oil carriers. The active ingredients are incorporated in water and oil phases.

Group 1 (VE) was treated with only vehicle, group 2 (VECH) with vehicle and chito-oliogosaccharids (2%), group 3 (VECA) with vehicle and carprofen (5.6%, total dose 0.5 – 0.6 mg/kg), group 4 (VECHCA) with vehicle, chito-oligosaccharids (2%) and carprofen (6.7 ± 15%, total dose 0.5 – 0.6 mg/kg), group 5 (S) was transcutaneously sham treated and group 6 (CA) was intravenously treated with carprofen (5%). This group received once per day 4 mg/kg BW carprofen intravenously (Rimadyl®, Pfizer AG, Zürich, Switzerland) during 4 days.

**Figure 1:**
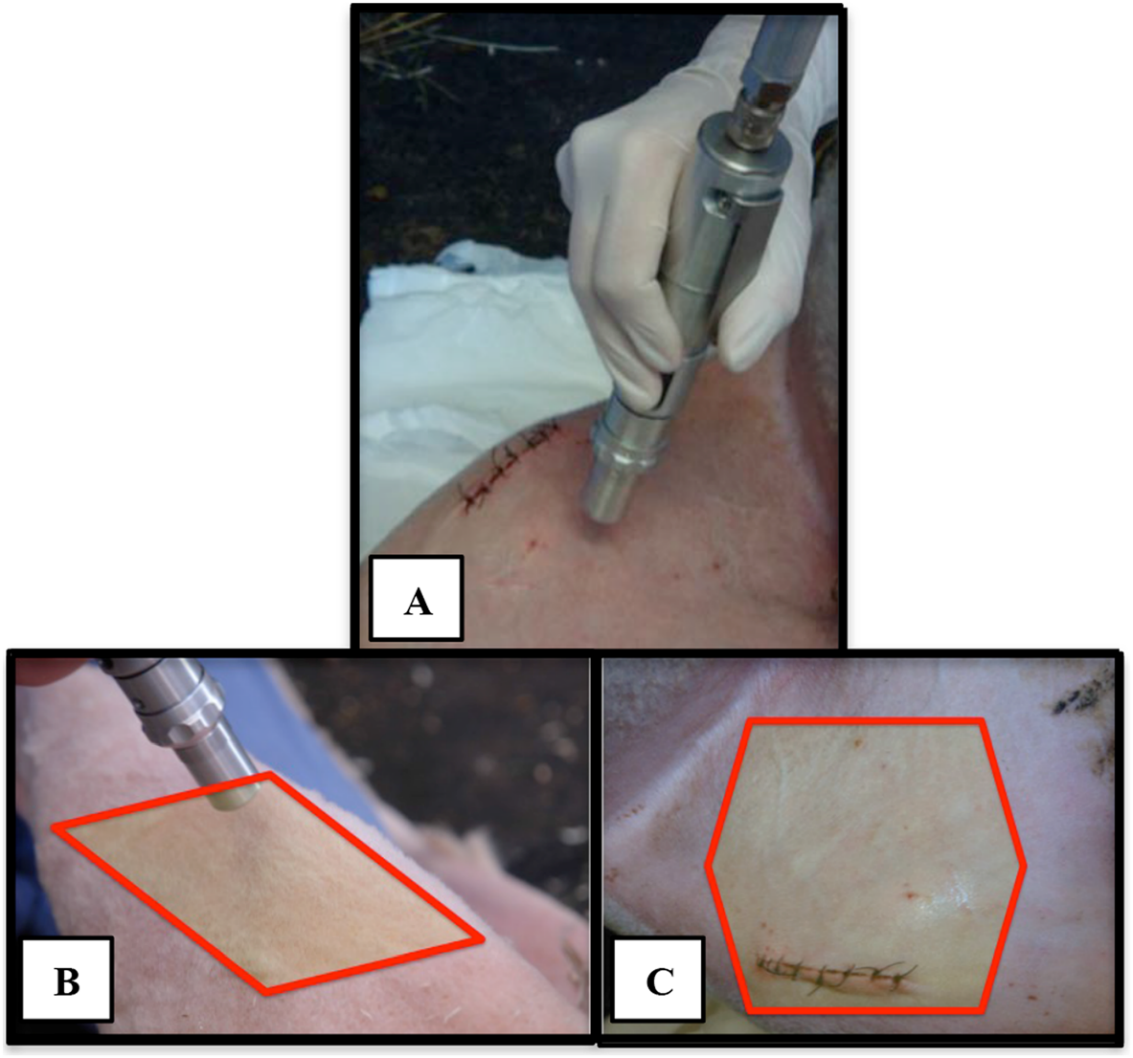
**Treatment procedure. A**. The applicator was held in a 90° angle to the application site. **B**. An area of approximately 10 cm2 was treated. **C**. Treatment area.

The transcutaneously treated groups were subjected to 18 applications, 3 applications per week at 15 minutes each for a total of six weeks (Figure 1). For drug delivery, the applicator was held in a distance of approximately 1 cm from the skin in an angle of 90° degrees. The areas of skin around the stifle joint were treated in an area of approximately 10 cm^2^ on the medial side of the limb, medial to the surgical wound and an equally sized area lateral to the surgical wound.

For the measurements of carprofen concentration presented here, 2 sheep from groups 3 (VECA), 4 (VECHCA) and 6 (CA) were included. The first transcutaneous treatment was performed one day prior to surgery (Day 0). The day of surgery was termed day 1. The transcutaneous treatments were performed on days 0, 3, 5, 8, 10, 12, 15, 17, 19, 22, 24, 26, 29, 31, 33, 36, 38 and 40. After the first two transcutaneous treatments (day 0 and 3) the blood samples were taken from groups 3 and 4 at −5, 15, 20, 30, 60 minutes and 2, 3, 6 and 18 hours in relation to the application. In addition, blood samples were taken on days 5, 12, 19, 26, 33 and 40, always 6 hours after the transcutaneous application.

**Figure 2:**
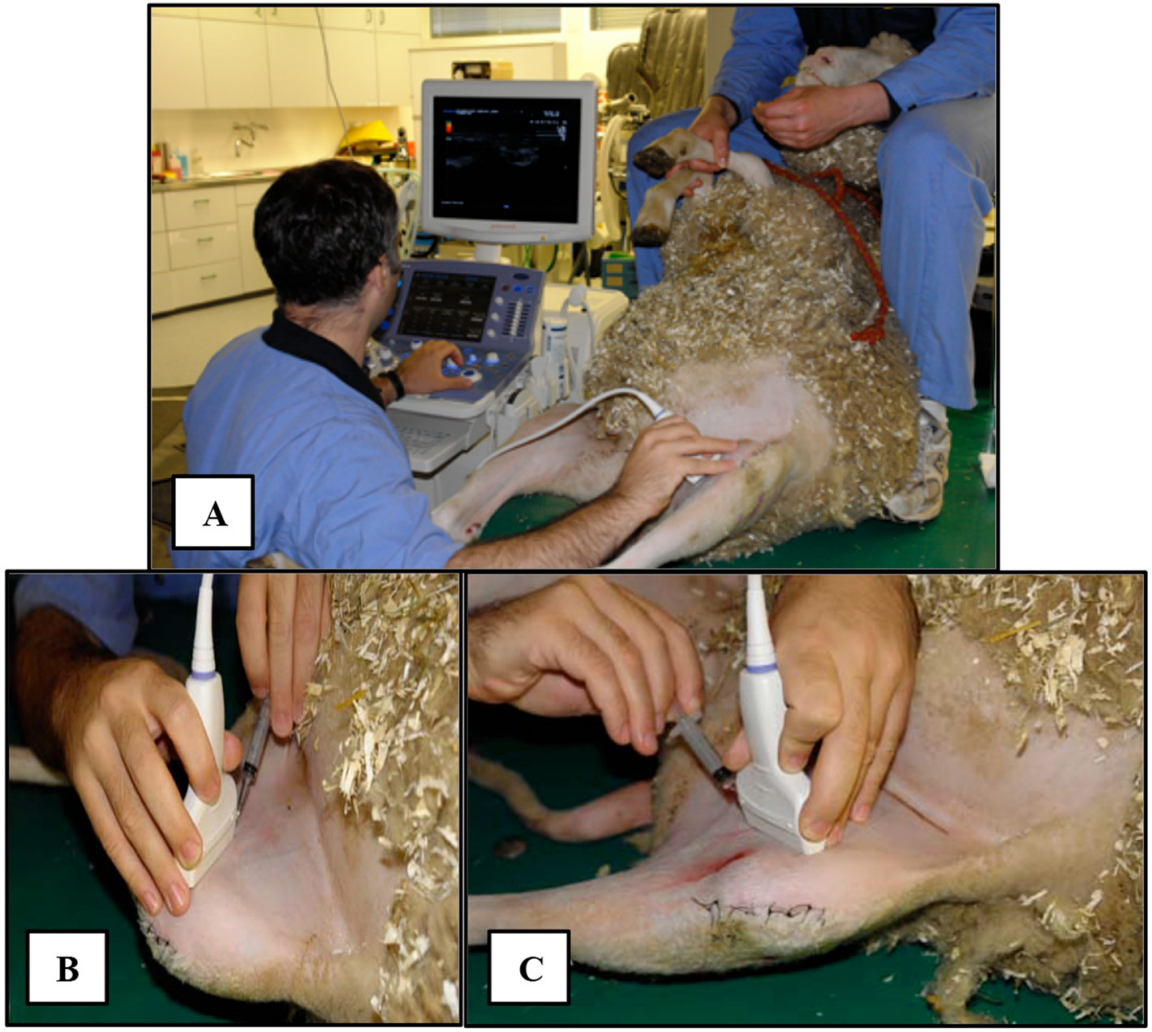
**US performance for synovial fluid sample taking. A**. Positioning of sheep. **B**. Setting how synovial fluid was taken. **C**. Position of the sheep's limb to expose the medial articular pocket.

Synovial fluid was collected during surgery and afterwards once a week during 6 weeks using ultrasound guidance (Figure 2). For this purpose the sheep were sedated with Medetomidine (20ug/kg BW, Dorbene, Dr. E. Graeub AG, Switzerland). They were placed on their hindquarters and positioned in a more lateral position in order to expose the medial articular pocket of the limb that underwent surgery.

Additionally, blood samples were taken for comparison. The blood samples from the intravenously treated group 6 (CA) were taken on day 1, 2, 3, 4, and 5 at time points −5, 10, and 60 minutes and 3, 6 and 12 hours after the iv. carprofen administration. Two further blood samples were taken on day 8 and 12.

All blood samples were taken from the jugular vein. The first 4 ml of blood was discarded and the remaining 6 ml of blood was filled into a lithium heparin tube and centrifuged for 20 minutes at 1500 U/min. The resulting plasma was transferred into labelled sterile 1.5 ml eppendorf tubes and stored at −20°C for batch analysis.

Synovial fluid samples were obtained using a 5 ml syringe (Braun, Omnifix®) and a 18 G needle (Terumo®, Terumo Europe N.V., Belgium) and were stored in pre-labelled sterile 1.5 ml eppendorf tubes. Each sample was wrapped in aluminium foil and stored at −20°C for later batch analysis (Interlabor Belp AG, Belp, Switzerland). The carprofen concentrations were measured using high performance liquid chromatography (HPLC) in connection with Tandem - Mass Spectroscopy (HPLC-MS/MS). The liquid chromatography (Dionex UltiMate 300, Software Dionex Chromatography MS Link Vers. 2.7.0.2251) was used to separate the mixture of compounds into single components and the Mass-Spectroscopy (Triple Quadrupol Sciex API 4000, Software Analyst Vers. 1.5.1) to identify and quantify the components.

## Results

### Group 3 (VECA, animals 5203/04)

**Figure 3:**
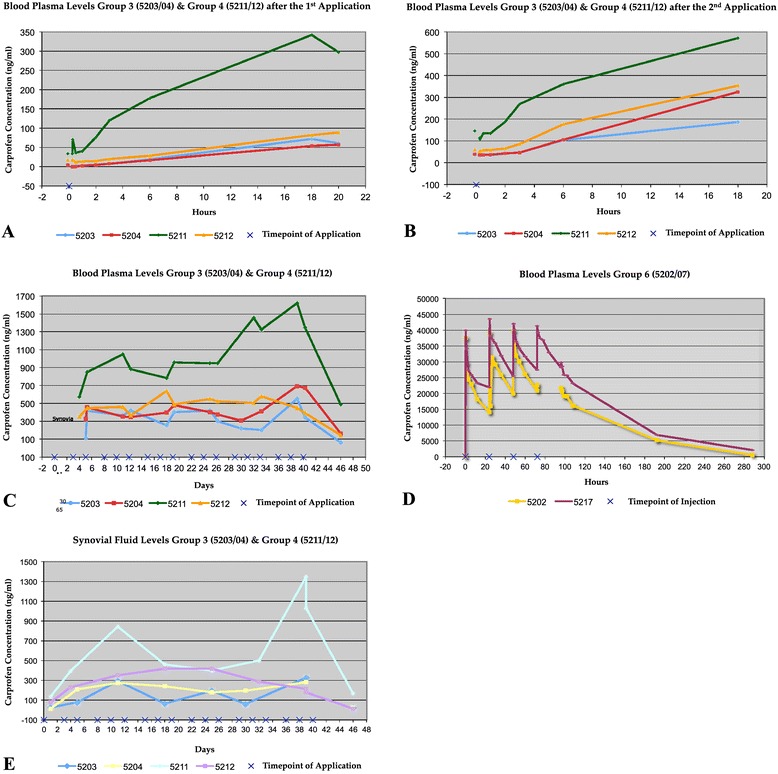
**Carprofen plasma and synovial fluid levels.** Overview of the blood plasma **(A-C)** and synovial fluid **(E)** carprofen levels of the transcutaneous treated groups 3 & 4 and the blood plasma levels of the intravenous treated group 6 **(D)**.

The first measurable carprofen values after the initial transcutaneous application were obtained at 30 min and 60 min in animals 5203 and 5204, respectively (<2 ng/ml) (Table 1). The highest values after the first application were measured after 18 hours (5203) and 20 hours (5204), respectively. Just before the second application (after 72 h), the blood concentration dropped to 49% (5203) and 67% (5204), respectively (Figure 3A).

**Table 1:** **Carprofen concentrations of plasma samples Group 3 (VECA)**

**Timepoint**	**5203 (VECA) c(ng/ml)**	**5204 (VECA) c(ng/ml)**
Day 0 (1 day prior surgery)	-	-
15 min. after 1^st^ application (day 0)	-	-
20 min. after 1^st^ application (day 0)	-	-
30 min. after 1^st^ application (day 0)	<2.0	-
1 h after 1^st^ application (day 0)	2.6	<2.0
2 h after 1^st^ application (day 0)	5.5	4.1
3 h after 1^st^ application (day 0)	7.2	7.7
6 h after 1^st^ application (day 0)	19.1	16.8
18 h after 1^st^ application (day 1)	72.0	54.1
Intra OP (day 1)	61.0	57.1
5 min. prior to 2^nd^ application (day 3)	35.4	38.0
15 min. after 2^nd^ application (day 3)	34.3	34.6
20 min. after 2^nd^ application (day 3)	33.8	35.4
30 min. after 2^nd^ application (day 3)	34.8	35.5
1 h after 2^nd^ application (day 3)	33.8	36.2
2 h after 2^nd^ application (day 3)	41.2	42.1
3 h after 2^nd^ application (day 3)	46.8	46.4
6 h after 2^nd^ application (day 3)	104	105
18 h after 2^nd^ application (day 4)	187	325
6 h after application (week 1)	416	456
6 h after application (week 2)	419	347
6 h after application (week 3)	402	479
6 h after application (week 4)	301	376
6 h after application (week 5)	203	412
6 h after application (week 6)	343	675
1 week after the last application (week 7)	63.4	163

After the second application, the blood values rose at one hour in both animals and the highest concentration was measured again after 18 hours in both animals. The peak concentration after the second application was 2.6- (5203) and 5.7- (5204) fold higher than the peak concentration after the first application (Figure 3B). The highest plasma level was measured in the 6th application week in both animals (553 ng/ml in 5203 and 695 ng/ml in 5204). After completion of the last application (week 6), the carprofen concentration in the plasma dropped. Six days after the last application the measured plasma level was not more than 18% (5203) and 24% (5204) of the highest value in week 6 (Figure 3C).

### Synovial samples

An overview of the synovial fluid measurements is provided in Table 2. The highest carprofen concentration in the synovial fluid was measured in week 6 and correlated with the highest carprofen plasma concentration. We measured values of 327 ng/ml (5203) and 281 ng/ml (5204), respectively (Figure 3E).

**Table 2:** **Carprofen concentrations of synovial fluid samples Group 3 (VECA)**

**Timepoint**	**5203 (VECA) c(ng/ml)**	**5204 (VECA) c(ng/ml)**
Intra OP (day 1)	25.3	13.4
Week 1 (taken under ultrasound control)	77.8	209
Week 2 (taken under ultrasound control)	293	274
Week 3 (taken under ultrasound control)	65.6	239
Week 4 (taken under ultrasound control)	195	178
Week 5 (taken under ultrasound control)	61.3	194
Week 6 (taken under ultrasound control)	327	281
Week 7 (1 week after the last application)	23.6	26.4

The synovial carprofen concentration was on average 48% (5203) and 49% (5204) lower than the simultaneously taken blood samples.

### Group 4 (VECHCA animals 5211/12)

#### Plasma samples

The highest concentrations after the first application were measured at 18 hours (5211) and 20 hours (5212), similarly to Group 3 (Table 3). After 72 hours, the carprofen concentration in the plasma dropped to 42% (5211) and 67% (5212) of the earlier measured peak plasma level (Figure 1A). After the second application, the highest concentration was measured in plasma of both animals at 18 hours. The peak concentration after the second application was 1.7- (5211) and 4- (5212) times higher than the peak concentration after the first application (Figure 3B).

**Table 3:** **Carprofen concentrations of plasma samples Group 4 (VECHCA)**

**Timepoint**	**5211 (VECHCA) c(ng/ml)**	**5212 (VECHCA) c(ng/ml)**
Day 0 (1 day prior surgery)	33.1	17.4
15 min. after 1^st^ application (day 0)	33.0	18.0
20 min. after 1^st^ application (day 0)	69.2	16.6
30 min. after 1^st^ application (day 0)	35.7	12.1
1 h after 1^st^ application (day 0)	40.5	13.7
2 h after 1^st^ application (day 0)	76.4	14.9
3 h after 1^st^ application (day 0)	120	20.0
6 h after 1^st^ application (day 0)	178	28.4
18 h after 1^st^ application (day 1)	342	81.9
Intra OP (day 1)	297	88.7
5 min. prior to 2^nd^ application (day 3)	145	59.6
15 min. after 2^nd^ application (day 3)	114	52.3
20 min. after 2^nd^ application (day 3)	108	53.1
30 min. after 2^nd^ application (day 3)	134	57.8
1 h after 2^nd^ application (day 3)	135	58.9
2 h after 2^nd^ application (day 3)	188	65.4
3 h after 2^nd^ application (day 3)	269	85.8
6 h after 2^nd^ application (day 3)	360	176
18 h after 2^nd^ application (day 4)	572	353
6 h after application (week 1)	849	448
6 h after application (week 2)	882	365
6 h after application (week 3)	959	490
6 h after application (week 4)	949	525
6 h after application (week 5)	1327	580
6 h after application (week 6)	1347	391
1 week after the last application (week 7)	486	146

The highest concentration of animal 5211 was measured in the 6th application week (1620 ng/ml). The highest concentration of animal 5212 was measured in the 3rd application week (635 ng/ml).

At 6 days after the last application the plasma concentration dropped to 36% (5211) and 37% (5212) of the peak values measured before (Figure 3C).

### Synovial samples

An overview of the measurements of the synovial fluid is provided in Table 4. The highest synovial concentration (1350 ng/ml) was measured simultaneously with the highest plasma concentration in the 6th application week in animal 5211. In animal 5212 the highest synovial concentration was measured in week 4 (418 ng/ml).

**Table 4:** **Carprofen concentrations of synovial fluid samples Group 4 (VECHCA)**

**Timepoint**	**5211 (VECHCA) c(ng/ml)**	**5212 (VECHCA) c(ng/ml)**
Intra OP (day 1)	134	78
Week 1 (taken under ultrasound control)	394	229
Week 2 (taken under ultrasound control)	845	352
Week 3 (taken under ultrasound control)	461	417
Week 4 (taken under ultrasound control)	398	418
Week 5 (taken under ultrasound control)	501	288
Week 6 (taken under ultrasound control)	1350	213
Week 7 (1 week after the last application)	165	13.4

The synovial concentration in week 7 reached 21% (5211) and 3% (5212) of the mean value of the afore measured peak concentrations (Figure 3E).

All synovial samples had lower carprofen concentrations than the plasma samples. The synovial samples reached 59% (5211) and 58% (5212) of the simultaneously taken plasma samples.

### Group 6 (CA animals 5202/17)

#### Plasma samples

The intravenously treated animals showed an interval peak plasma concentration, as described in a previous study [17] in sheep. The concentration was highest 10 minutes after the iv. application and lowest shortly before the next application (Table 5). The plasma concentration dropped after completion of the treatment. Nevertheless, after 12 days, the plasma level still presented with concentrations of 500 ng/ml (5202) and 2115 ng/ml (5217), respectively (Figure 3D).

**Table 5:** **Carprofen concentration measurements of plasma samples Group 6 (CA)**

**Timepoint**	**5202 (CA) c(ng/ml)**	**5217 (CA) c(ng/ml)**
Day 1 (day of surgery)	0.0	0.0
10 min. after 1^st^ injection (day 1)	37500	39950
1 h after 1^st^ injection (day 1)	29400	34217
2 h after 1^st^ injection (day 1)	24400	27300
3 h after 1^st^ injection (day 1)	23000	25917
12 h after 1^st^ injection (day 1)	18200	23317
5 min prior to 2^nd^ injection (day 2)	14100	21965
10 min after 2^nd^ injection (day 2)	39100	43583
1 h after 2^nd^ injection (day 2)	16000	38217
2 h after 2^nd^ injection (day 2)	31400	37068
3 h after 2^nd^ injection (day 2)	29200	35967
12 h after 2^nd^ injection (day 2)	25900	31900
5 min prior to 3^rd^ injection (day 3)	20000	25550
10 min after 3^rd^ injection (day 3)	39700	42083
1 h after 3^rd^ injection (day 3)	36100	40150
2 h after 3^rd^ injection (day 3)	31900	36400
3 h after 3^rd^ injection (day 3)	30100	34317
12 h after 3^rd^ injection (day 3)	26200	31650
5 min prior to 4^th^ injection (day 4)		27417
10 min after 4^th^ injection (day 4)		41300
1 h after 4^th^ injection (day 4)		39267
2 h after 4^th^ injection (day 4)		37433
3 h after 4^th^ injection (day 4)		36917
12 h after 4^th^ injection (day 4)		32950
1 day after the last injection (day 5)	16200	23150
3 days after the last injection (day 8)	5200	6917
1 week after the last injection (day 12)	500	2115

### Comparisons between groups 3, 4 and 6

The highest concentration of the intravenously treated group was measured at 10 minutes after the first application, whereas the highest concentration of the transcutaneously treated groups was measured after 18 – 20 hours.

After the iv. administration of carprofen, the plasma concentration reached an average of 38.7 μg/ml whereas the plasma level of the transcutaneously treated group recorded an average of 139.95 ng/ml. Therefore, the plasma concentration of carprofen in the transcutaneously treated animals (groups 3 VECA and 4 VECHCA) remained almost 300-fold lower than in the intravenously treated group 6 (CA). It should be noted however, that the overall carprofen dose was greater following intravenous injection as compared to transcutaneous. The ratio of measured plasma concentration to totally administered dose was as follows: in group 3, 1:80 (animal 5203) and 1:85 (animal 5204); group 4, 1:17 (animal 5211) and 1:65 (animal 5212).

The volume of distribution was low in the iv. group 6 (0.09 – 0.11 l/kg) shortly after the injection, but increased until the next injection. This was in contrast to the transcutaneously treated group, where the highest volumes of distribution were calculated shortly after the application (16.86 – 125 l/kg), and reduced over time.

## Discussion

In this study, we concentrate on reporting of the results of the carprofen receiving Groups 3 (VECA) and 4 (VECHCA) and show that the Vetdrop® System allows for transcutaneous delivery of carprofen. The results of the other 3 Groups are not addressed because they did not receive carprofen, but their results are reported elsewhere (Dissertation Nathalie Fouché, 2012).

In Groups 3 (VECA) and 4 (VECHCA) detectable carprofen concentrations were found in synovial fluid of the stifle as well as in the systemic blood circulation, although systemic concentrations were lower compared to control animals treated with carprofen intravenously.

The concentration of carprofen and some pharmacokinetic values could be determined, but direct comparisons with the control group (iv. application) were not considered totally appropriate. Pharmacokinetic calculations are based on the assumption that drugs are administered into a central compartment and subsequently distributed into peripheral compartments. This sequence is inverted with transcutaneous administrations and as such, pharmacokinetic algorithms may not fully apply in the case of transcutaneous delivery. Nevertheless, the standard pharmacokinetic formulas may help to interpret the data.

Factors, such as anatomical site, age, sex and skin disease influence the consistency of the epidermis and consequently the ability of substances to penetrate the skin barrier. Furthermore, drug delivery is also dependent on the pathways of skin permeation. Generally, it is considered that drug components can either pass the epidermis through a combination of transport routes including appendages or via a trans- or an intracellular route, and that the relative contribution of each of these is dependent on the physicochemical properties of the formulation and the properties of the skin.

Carprofen is a commonly used NSAID in veterinary medicine and was thus considered an ideal study material for the purposes of the current study. It has strong analgesic, antipyretic and antiphlogistic effects [17–19], although the exact mechanism of action of carprofen is not yet clear [20]. In vitro assays performed, using canine and equine chondrocytes demonstrated carprofen as having an inhibitory effect on prostaglandin-E2-synthesis [15,21].

In our study, both Groups 3 (VECA) and 4 (VECHCA) reached the peak plasma level (57.1 – 342 ng/ml) between 18 – 20 hours after the first application.

In a transdermally administered fentanyl patch (2.05 μg/kg/h) study in 21 sheep, Ahern et al. [22] observed peak plasma concentrations between 4 and 24 h (0.62 – 2.73 ng/ml). Although the peak plasma concentration levels in the current study were wide ranging, they were always reached.

After the 2^nd^ application, peak plasma level rose up to 5.7-fold higher (up to 572 ng/ml) as compared to the initial application. This indicated that carprofen accumulated in the body. Nevertheless, the measured plasma concentration was consistently lower as compared to the intravenous administration of carprofen (up to 42 μg/ml, 10 min after 3^rd^ application, = 48 h after the 1^st^ application).

The carprofen concentration in the synovial fluid of group 3 (VECA) reached between 48 – 49% of the carprofen plasma level and in group 4 (VECHCA) 58 -59%. Group 4 (VECHCA) accumulated noticeably more carprofen in the synovial fluid as compared to group 3 (VECA). This effect may have been related to the additional chito-oligosaccharids in group 4 (VECHCA).

Analysing transdermally administered pharmaceutical agents raises the question as to whether the active ingredients reach their target location through direct penetration of the target tissue or through redistribution from systemic blood circulation Mills et al. [23] stated that topically applied NSAIDs can penetrate into deeper tissues and into the synovial fluid, and that local blood redistribution is contributing to this effect. They suggested that certain NSAIDs might increase the local blood flow and assist in transporting the applied drugs deeper into the tissue prior to their distribution into the systemic blood circulation. Indeed, Singh and Roberts [24] demonstrated that local tissue penetration of NSAIDs was possible up to a depth of 3 – 4 mm. However, Radermacher et al. [25] observed that the distribution of topically applied diclofenac into the synovial fluid was achieved through the blood supply and that direct penetration was minimal.

## Conclusions

In this preliminary study we showed that the measured synovial concentration was always lower than the plasma concentration. Nevertheless, carprofen reached concentrations in the joint that are considered as being admissible for long-term therapy. Whether carprofen is transported directly into the joint or redistributed after systemic circulation is not yet clear and further investigations are needed. Therefore, in a main study a larger pharmacokinetic analysis needs to be set up.
